# Forebrain Lineage Factor DMRTA2 Is Not Required for Maintenance of H3G34‐Mutant Paediatric High‐Grade Gliomas

**DOI:** 10.1111/jcmm.71302

**Published:** 2026-07-25

**Authors:** Raul B. Bressan, Oliva Sanchez, Carlos Alberto Oliveira de Biagi‐Junior, Raúl Torres‐Ruiz, Gustavo Alencastro Veiga Cruzeiro, Irene Salgado, Sandra Rodriguez‐Perales, Eduardo Caleiras, Zaira Vega, Gillian Morrison, Steven M. Pollard, Mariella G. Filbin, Manuel Valiente

**Affiliations:** ^1^ Spanish National Cancer Research Centre Madrid Spain; ^2^ Dana‐Farber/Boston Children's Cancer and Blood Disorders Center Harvard Medical School Boston Massachusetts USA; ^3^ Division of Hematopoietic Innovative Therapies, Biomedical Innovation Unit Centro de Investigaciones Energéticas, Medioambientales y Tecnológicas Madrid Spain; ^4^ Advanced Therapies Unit Instituto de Investigación Sanitaria Fundación Jiménez Díaz Madrid Spain; ^5^ Centro de Investigación Biomédica en Red de Enfermedades Raras Madrid Spain; ^6^ Centre for Regenerative Medicine, Institute for Regeneration and Repair University of Edinburgh Edinburgh UK; ^7^ Cancer Research UK Scotland Centre Edinburgh UK

## Abstract

Developmental transcriptional programs are increasingly recognized as key drivers of paediatric gliomagenesis. Here, we unexpectedly find that the forebrain‐specific doublesex‐ and mab‐3‐related transcription factor a2 (DMRTA2), despite being highly expressed in H3G34‐mutant diffuse hemispheric glioma (DHG‐H3G34) and implicated in tumour initiation, is not required for the maintenance of established patient‐derived tumour cells. These findings support a stage‐specific framework in which transcriptional dependencies are dynamically rewired during tumour progression in paediatric brain tumours.

Diffuse hemispheric gliomas, H3G34R/V‐mutant (DHG‐H3G34), are highly aggressive paediatric high‐grade gliomas that arise exclusively in the cerebral hemispheres and are thought to originate from interneuronal progenitors of the developing forebrain [1, 2, 3]. Despite recent advances in molecular characterization, these tumours remain poorly understood and lack effective targeted therapies. Using isogenic cellular models of DHG‐H3G34, our previous work indicates that, in contrast to other oncohistones, H3.3‐G34R does not impose widespread transcriptional or epigenetic changes. Instead, it promotes focal stabilization of highly expressed genes, particularly those associated with forebrain progenitor identity, thereby reinforcing pre‐existing transcriptional networks and locking initiating forebrain cells into an immature, proliferative state [4].

To identify candidate transcriptional mediators downstream of H3.3‐G34R, we analysed transcriptomic profiles from three independent patient‐derived isogenic models in which the mutant allele had been corrected or ablated using CRISPR‐based gene editing [4, 5]. This analysis identified a small set of transcription factors consistently downregulated upon loss of H3.3‐G34R (Figure 1a), suggesting their potential role as downstream effectors of the oncogenic program. Among these, *DMRTA2*—a transcription factor that plays a key role in progenitor maintenance during cortical development [6, 7] emerged as the most promising candidate (Figures 1a,b, S1a). Of note, its expression was also significantly increased in an engineered foetal neural stem cell DHG model based on ectopic induction of H3.3‐G34R [4, 8] (Figure.S1b) and was consistently enriched in DHG‐H3G34 tumours compared with both H3‐wild‐type gliomas and H3K27M‐mutant diffuse midline gliomas across multiple patient cohorts (Figures 1c, S1c).

**FIGURE 1:**
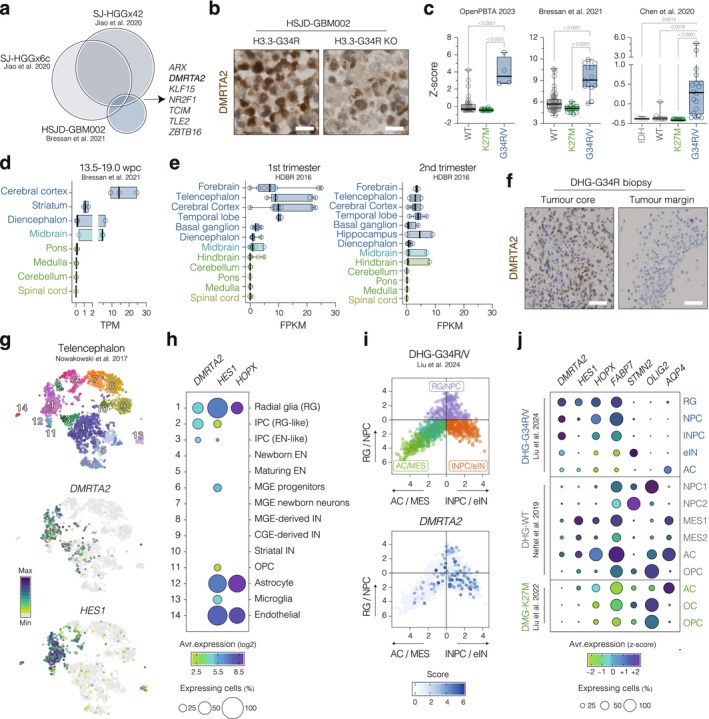
DMRTA2 is enriched in human forebrain progenitors and H3G34‐DHGs. (a) Venn diagram showing overlapping genes downregulated upon H3.3‐G34R correction across independent isogenic patient‐derived models (SJ‐HGGx42, SJ‐HGGx6c and HSJD‐GBM002). Highlighted are seven transcription factor genes consistently downregulated across all three models. (b) Immunohistochemical analysis confirming reduction in DMRTA2 levels in H3.3‐G34R knockout HSJD‐GBM002 cells. Scale bar, 20 μm. (c) Bar plots showing *DMRTA2* expression across paediatric high‐grade glioma cohorts, highlighting increased levels in H3G34R/V tumours relative to hemispheric H3‐wild‐type and midline H3K27M tumours. Statistical significance was determined using one‐way ANOVA with Tukey's multiple comparisons test. (d,e) Bar plots showing *DMRTA2* expression across human brain regions during development, highlighting enrichment in forebrain/telencephalic regions during the first gestational trimester. Developmental stages and data sources are indicated. (f) Immunohistochemical staining of DMRTA2 in a DHG‐H3G34R patient biopsy confirms strong expression in the tumour core and absence in histologically normal cells at the tumour margin. Scale bar, 50 μm. (g) t‐SNE projection of single‐cell RNA‐seq data from the developing human telencephalon (13), showing enriched expression of *DMRTA2* and the progenitor marker *HES1* across the cell clusters. (h) Dot plot showing *DMRTA2* expression across cell clusters indicated in panel g, highlighting enrichment in radial glia and progenitor populations marked by *HES1* and *HOPX* expression. Circle size indicates the percentage of positive cells, and colour intensity reflects the average expression levels in positive cells. (i) Projection of *DMRTA2* expression onto a two‐dimensional representation of lineage‐state scores derived from scRNA‐seq data of H3G34R/V gliomas, as defined in (3). The y‐axis represents stem‐like radial glia/neural progenitor cell (RG/NPC)‐like scores, whereas the x‐axis represents differentiated intermediate neuronal progenitor/excitatory neuron‐like (INPC/eIN)‐like scores. Analogous to the developing human brain, *DMRTA2* expression is enriched in progenitor‐like cellular states. (j) Dot plot showing expression of DMRTA2 and lineage marker across cellular states identified in DHG‐H3G34, adult H3‐wild‐type DHG and DMG‐H3K27 single cell transcriptomics as defined in (3).

Consistent with the anatomical distribution of DHG‐H3G34, analysis of DMRTA2 expression across human tissues and developmental stages revealed a highly restricted pattern, with prominent expression in the forebrain during foetal development, particularly within the first trimester (Figures 1d,e, S1d). In agreement, immunohistochemical analysis of a DHG‐H3G34 biopsy confirmed strong nuclear DMRTA2 staining within regions of high tumour cellularity, while adjacent histologically normal areas lacked detectable expression (Figures 1f, S1e), indicating tumour‐associated maintenance of this developmental transcription factor.

Further analysis of independent single‐cell RNA sequencing (scRNA‐seq) datasets from the developing human brain confirmed that *DMRTA2* expression is enriched within forebrain structures, particularly the telencephalon and is largely restricted to progenitor populations, including radial glial cells (Figures 1g,h, S1f–i). These findings are consistent with recent work demonstrating robust expression of *DMRTA2* in radial glia and its role in maintaining progenitor identity during development and early disease organoid‐based models [9]. Similarly, interrogation of paediatric high‐grade glioma scRNA‐seq datasets revealed that *DMRTA2* expression is enriched in radial glia‐ and intermediate progenitor‐like tumour cell populations in DHG‐H3G34, whereas minimal expression was observed in H3‐wild‐type and H3K27M‐mutant paediatric gliomas (Figure 1i,j).

Given these observations and the recently reported role of DMRTA2 in regulating radial glial maintenance and tumorigenicity [9], we next sought to determine whether DMRTA2 is required for the continued malignant behaviour of fully transformed tumour cells. To this end, we utilized a panel of patient‐derived glioma stem cell cultures harbouring H3G34R/V mutations (Figure 2a). Immunohistochemical analyses confirmed that the developmental expression pattern of DMRTA2 is faithfully recapitulated in these models, with strong nuclear DMRTA2 expression across G34‐mutant cell lines (Figure 2a).

**FIGURE 2:**
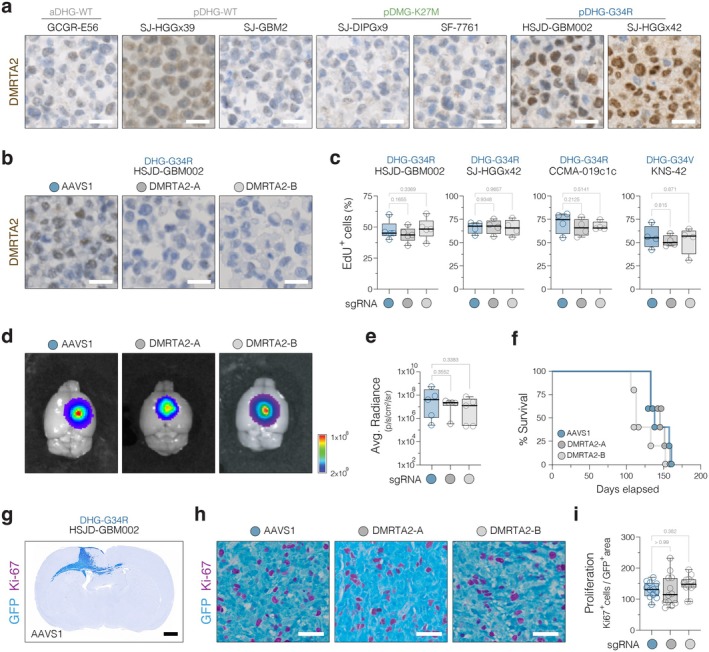
DMRTA2 is not required for proliferation and tumour‐forming capacity of patient‐derived H3G34‐DHG cells. (a) Immunohistochemical analysis of high‐grade glioma patient‐derived cell lines from distinct subtypes shows increased DMRTA2 expression in H3G34‐mutant cells. (b) Immunohistochemical staining confirms loss of DMRTA2 protein following lentiviral transduction with Cas9 and sgRNAs targeting the DMRTA2 gene. An AAVS1‐targeting sgRNA was used as a control. HSJD‐GBM002 cells are shown as a representative example. Scale bar, 20 μm. (c) Quantification of EdU incorporation in multiple H3G34R/V glioma cell lines following DMRTA2 knockout demonstrates no significant effect on proliferation. Data represent mean ± standard deviation. Each dot represents an independent experiment. Statistical significance was assessed using one‐way ANOVA followed by Dunnett's multiple comparisons test, comparing each DMRTA2 knockout to its corresponding AAVS1 control. (d) Representative bioluminescence images of orthotopic xenografts generated from control and DMRTA2 knockout HSJD‐GBM002 cells. (e) Quantification of in vivo tumour burden by bioluminescence imaging in HSJD‐GBM002 orthotopic xenografts 3 months after intracranial transplantation. Data represent mean ± standard deviation (*n* = 5 mice per group). Statistical significance was assessed using one‐way ANOVA followed by Dunnett's multiple comparisons test, comparing each DMRTA2 knockout group with the AAVS1 control. (f) Kaplan–Meier survival curves of mice bearing orthotopic HSJD‐GBM002 xenografts following DMRTA2 knockout. No significant differences in overall survival were observed between control and knockout groups. Statistical significance was assessed using the log‐rank (Mantel–Cox) test. *n* = 5 mice per group. (g‐h) Representative GFP/Ki67 immunohistochemical staining of orthotopic xenografts derived from control (sgAAVS1) and DMRTA2 knockout HSJD‐GBM002 cells. Scale bars: 1 mm and 50 μm, respectively. (i) Quantification of Ki67‐positive tumour cells in HSJD‐GBM002 orthotopic xenografts. Data represent mean ± standard deviation. Each dot represents one field of view from an individual mouse. Statistical significance was assessed using the Kruskal–Wallis test followed by Dunn's multiple comparisons test.

Using CRISPR/Cas9‐mediated gene editing, we generated DMRTA2‐deficient derivatives of four independent patient‐derived H3G34R/V glioma cell lines. Editing of the DMRTA2 locus was confirmed by PCR amplification and Sanger sequencing of the targeted region, with sequence chromatograms demonstrating mixed nucleotide peaks immediately downstream of the predicted sgRNA cleavage site, indicative of indel formation (Figure S2a). Furthermore, immunohistochemistry confirmed loss of DMRTA2 protein expression in CRISPR‐edited cells (Figures 2b, S2b).

Notably, in contrast to its recently reported role in developmental and engineered models of H3G34 gliomagenesis [9], loss of DMRTA2 did not result in overt morphological changes or impaired proliferation as assessed by EdU incorporation assays (Figure 2c). These findings were consistent across all tested models, suggesting that DMRTA2 is not required to sustain proliferation of genetically complex, established tumour cells.

To further evaluate the functional relevance of DMRTA2 in vivo, we performed orthotopic intracranial transplantation assays with the H3G34R‐mutant cell line HSJD‐GBM002. Notably, *DMRTA2* knockout did not significantly affect tumour growth or overall survival of tumour‐bearing animals (Figure 2d–f). Histological analysis of xenograft tumours revealed no significant differences in the expression of the proliferation marker Ki67 between control and knockout conditions (Figure 2g–i), further supporting that DMRTA2 is not essential for tumour maintenance in established disease contexts. Similar results were obtained with an independent mutant line (SJ‐HGGx42, Figure S2c–e).

Altogether, these findings build upon the recent work of Royston et al. [9], who identified DMRTA2 as a key regulator of radial glial maintenance and tumour initiation using genetically engineered models that recapitulate the earliest stages of H3G34 gliomagenesis. Extending these observations to patient‐derived models of established disease, we find that DMRTA2 is no longer required for tumour maintenance, suggesting that its functional requirement is temporally restricted. Whereas these early developmental models rely on a limited number of initiating oncogenic events, established patient tumours acquire numerous additional genetic and epigenetic alterations during disease evolution [1, 2, 10]. Such progressive molecular diversification is likely to remodel the transcriptional networks that sustain the malignant state, potentially diminishing the requirement for developmental regulators that are critical during tumour initiation.

This interpretation is consistent with the emerging view that oncogenic dependencies evolve throughout tumour progression rather than remaining static [11]. Developmental transcription factors that facilitate lineage specification or malignant transformation may become functionally redundant as tumour cells accumulate cooperating alterations that reinforce progenitor identity and proliferative capacity through alternative regulatory mechanisms [11, 12]. In keeping with this concept, developmental transcription factors involved in corticogenesis operate within highly interconnected regulatory networks [13], raising the possibility that compensatory activation of related transcriptional programs may preserve lineage identity following DMRTA2 loss. Future transcriptomic profiling of DMRTA2‐deficient tumours may provide mechanistic insights into the downstream regulatory programs controlled by DMRTA2 and identify adaptive mechanisms that emerge during the transition from tumour initiation to tumour maintenance.

The possibility that DMRTA2 contributes to additional context‐dependent functions beyond tumour maintenance also warrants further investigation. Future studies should determine whether DMRTA2 contributes to additional biological processes, including cell fitness, lineage plasticity and therapeutic response, particularly under biological contexts that challenge cellular identity or expose latent developmental dependencies. Importantly, these findings do not imply that all components of the forebrain developmental program become functionally redundant during tumour progression. Previous studies have demonstrated that regulators such as FOXG1 and CDK6 [3, 4] remain essential for tumour maintenance in patient‐derived H3G34 glioma models, indicating that selected elements of the cell‐of‐origin transcriptional network are retained as therapeutic vulnerabilities after malignant transformation. Together, these findings demonstrate that developmental transcriptional dependencies in gliomagenesis can be stage‐specific and highlight the importance of distinguishing regulators required for initiation from those that remain actionable therapeutic vulnerabilities in established disease.

## 1 Material and Methods

### 1.1 Analysis of Publicly Available Bulk Transcriptomic Datasets

Processed count data for patient tumour samples were obtained from the Supporting Information of Chen et al., 2020 [2] and Bressan et al., 2021 [4]. For the OpenPBTA cohort, normalized expression (z‐score) values were retrieved from the paediatric cBioPortal (https://pedcbioportal.org). For isogenic patient‐derived cell pairs, differential expression analysis was performed using GEO2R on datasets GSE138077 and GSE163044, with samples grouped according to H3.3‐G34R status. Significance was defined as adjusted *p* < 0.05 and log_2_ fold change > 1. Expression values from human foetal brain regions were retrieved from NCBI GEO (GSE163044) [4] and the EMBL‐EBI Expression Atlas (HDBR, E‐MTAB‐4840). Expression values across organs and developmental stages were obtained from the Evo‐devo mammalian database [14].

### 1.2 Analysis of Publicly Available Single‐Cell RNA‐Seq Datasets

Publicly available, pre‐processed scRNA‐seq datasets of human brain development [15, 16, 17] were analysed using the UCSC Cell Browser. scRNA‐seq datasets from glioma patient samples were analysed as previously described [3].

### 1.3 CRISPR Vector Production

sgRNAs were designed using the Benchling CRISPR gRNA Design Tool and cloned into lentiCRISPRv2 (Addgene #52961). Lentiviral production was performed as previously described [18]. Generation of indels at sgRNA was confirmed by PCR followed by Sanger sequencing. Primer and sgRNA sequences are listed in the Table S1.

### 1.4 Cell Culture

All cell lines were maintained as standardized monolayers in serum‐free medium supplemented with N2 and B27, laminin‐1 (1 μg/mL) and growth factors EGF and basic FGF (10 ng/mL each), as previously described [4]. Medium was refreshed twice weekly and cells were passaged at a ratio of 1:3–1:5 upon reaching confluence. For generation of DMRTA2 knockout lines, supernatant from lentiviral‐producing HEK293 cells was collected, filtered and used to transduce glioma cell lines in the presence of polybrene (5 μg/mL) for 24 h. Transduced cells were selected with puromycin (2 μg/mL) for 96 h, until complete cell death was observed in non‐infected control cultures.

### 1.5 Immunohistochemistry

Brain tissue and cell pellets were collected and fixed in 4% PFA solution overnight. Following paraffin embeding, 10 μm slices were sectioned, deparaffinized and rehydrated by a graded ethanol series to water. Antigen retrieval was performed using PT link high buffer for DMRTA2 and Ventana/CC1 for GFP/KI67 double staining. Sections were incubated with primary antibodies against DMRTA2 (#HPA062958–1/50, 60 min), GFP (#11814460001, 1/500, 16 min) and Ki‐67 (#IR626, 36 min) Immunohistochemical reactions were developed using ChromoMap DAB, DISCOVERY Purple or Teal Kit (Roche/Ventana). Nuclei were counterstained with haematoxylin. Slides were then counterstained with haematoxylin, dehydrated and mounted for microscopic evaluation on a Axio Scan.z1 slide scanner. All procedures were performed on an automated immunostaining pla4orms (Autostainer Link 48, Agilent; Discovery XT‐ULTRA, Roche/Ventana).

### 1.6 Edu Incorporation Assay

Cells were plated at 5000 cells/cm^2^ in 48‐well plates and cultured for 72 h, followed by incubation with 10 μM EdU for 24 h. EdU staining was performed using the Click‐iT EdU assay kit. Positive cells were quantified across at least ten random fields per experiment, with the total cell number determined by DAPI staining.

### 1.7 Intracranial Transplantation

For in vivo tumour growth experiments, 2 μL of cell suspension (100,000 cells/μL) was stereotactically injected into the right frontal cortex of 6–8‐week‐old nude mice (approximately 1.5 mm lateral and 1 mm caudal to bregma, at a depth of 2 mm) under general anaesthesia. For bioluminescence imaging, D‐luciferin (50 mg/kg) was administered retro‐orbitally, and animals were imaged using an IVIS Lumina LT Series III system (PerkinElmer). Animals were monitored until they became moribund or developed severe neurological symptoms, at which point they were euthanized and intracranial tumour formation was confirmed under a fluorescent stereoscope. All procedures were performed in accordance with protocols approved by the institutional ethics board and the Animal Care and Use Committee of Comunidad de Madrid (PROEX 042.2–25).

### 1.8 Statistical Analysis

Statistical analyses were performed using GraphPad Prism. Data were tested for normality using the Shapiro–Wilk test. For normally distributed data, comparisons were performed using Student's *t*‐test (two groups) or one‐way ANOVA (three or more groups). For non‐normally distributed data, comparisons were performed using the Kruskal–Wallis test. *p* Values are indicated in the figure panels.

## Author Contributions

**Raul B. Bressan:** conceptualization, investigation, funding acquisition, writing – original draft, methodology, data curation, writing – review and editing, visualization, project administration, supervision, formal analysis. **Sandra Rodriguez‐Perales:** resources. **Oliva Sanchez:** data curation, investigation. **Manuel Valiente:** supervision, funding acquisition, writing – review and editing, project administration. **Eduardo Caleiras:** investigation. **Zaira Vega:** investigation. **Raúl Torres‐Ruiz:** resources. **Carlos Alberto Oliveira de Biagi‐Junior:** formal analysis, data curation, visualization. **Gillian Morrison:** resources. **Mariella G. Filbin:** resources, supervision. **Steven M. Pollard:** resources. **Irene Salgado:** investigation. **Gustavo Alencastro Veiga Cruzeiro:** data curation, formal analysis.

## Funding

This work was supported by Agencia Estatal de Investigación, RYC2024‐048827‐I. Ministerio de Ciencia, Innovación y Universidades, FPU22/03421, PI23/01932, PID2021‐124582OB‐I00. Fundación Científica Asociación Española Contra el Cáncer, PRYGN259169VALI, PRYCO234528VALI, TRNSC213878VALI. Fundacion la Caixa, HR23‐00051. Instituto de Salud Carlos III, AC20/00114.

## Conflicts of Interest

M.V. reports a research agreement with AstraZeneca. S.M.P. is founder and shareholder of Trogenix, which is developing novel gene therapies for brain tumours. The remaining authors declare no competing interests.

## Supporting information


**Figure S1:** Extended characterization of DMRTA2 expression across developmental and tumour contexts. (a) Heatmaps showing log_2_ fold‐change of candidate transcription factors following H3.3‐G34R correction in three independent patient‐derived isogenic H3G34‐mutant glioma models (HSJD‐GBM002, SJ‐HGGx6c and SJ‐HGGx42), highlighting DMRTA2 among the shared downregulated genes. (b) Heatmaps showing log_2_ fold‐change of the same candidate transcription factor genes in an engineered forebrain neural stem cell model (4) harbouring TP53 knockout (TP53‐KO), PDGFRA overexpression (PDGFRA‐OE) and either wild‐type or G34R‐mutant H3.3 constructs. (c) Z‐score expression of the seven candidate genes across WT, H3K27M and H3G34R/V paediatric high‐grade gliomas (2). Bars represent mean ± standard deviation. Statistical significance was assessed using one‐way ANOVA followed by Tukey's multiple comparisons test. Pairwise comparisons are indicated. n.s., not significant. (d) Scatter plots showing DMRTA2 expression across human tissues and developmental stages (12), demonstrating enrichment in the foetal forebrain relative to other organs. Horizontal dashed lines indicate birth. (e) Representative immunohistochemistry for DMRTA2 in a brain metastasis control and in a DHG‐G34R tumour biopsy. (f) 2D projection of first‐trimester human brain single‐cell RNA‐seq dataset (15), annotated by region and lineage, with DMRTA2 and HES1 expression overlaid. (g) Quantification of the percentage of DMRTA2‐expressing cells across first‐trimester brain regions and lineages as shown in panel f. (h) 2D projection of second‐trimester cortical single‐cell RNA‐seq dataset [14], annotated by region and lineage, with *DMRTA2* and *HES1* expression overlaid. (i) Quantification of the percentage of *DMRTA2*‐expressing cells across cortical regions and cellular lineages as shown in panel h.

**Figure S2:** Extended characterision of DMRTA2 knockouts in patient derived DHG‐G34 cell lines. (a) Schematic of the *DMRTA2* locus showing the position of the CRISPR/Cas9 sgRNA target sites, PCR amplification and Sanger sequencing primers. Blue arrowheads indicate the predicted Cas9 cleavage sites. Representative forward and reverse Sanger sequencing chromatograms from sgDMRTA2‐A‐ and sgDMRTA2‐B‐transduced cells demonstrate mixed nucleotide peaks arising immediately downstream of the predicted Cas9 cleavage sites, consistent with CRISPR‐mediated indel formation at the targeted locus. (b) IHC analysis confirming loss of DMRTA2 protein expression in SJ‐HGGx42 cells transduced with DMRTA2 shRNA‐B. Scale bar, 20 μm. (c) Representative bioluminescence imaging of orthotopic xenografts derived from SJ‐HGGx43 cells transduced with sgAAVS1 control and sgDMRTA2‐B. (d) Quantification of in vivo tumour burden by bioluminescence imaging in HSJD‐GBM002 orthotopic xenografts 1 month after intracranial transplantation. Data represent mean ± standard deviation. Statistical significance was assessed using an unpaired two‐tailed Student's *t*‐test with Welch's correction. *n* = 4 mice per group. (e) Right, Kaplan–Meier survival analysis of tumour‐bearing mice. μm. *n* = 4 animals per group.

**Table S1:** Sequences of CRISPR sgRNAs and genotyping primers used in the study.

## Data Availability

Unique reagents generated in this study are available from the corresponding author upon request. Published transcriptomic datasets used in the study are openly available as indicated in the material and methods section.
