# Supplementary figures and images for: Forebrain Lineage Factor DMRTA2 Is Not Required for Maintenance of H3G34‐Mutant Paediatric High‐Grade Gliomas
Source: J Cell Mol Med. 2026 Jul 25;30(14):e71302. doi: 10.1111/jcmm.71302 (PMC13401718; doi:10.1111/jcmm.71302)

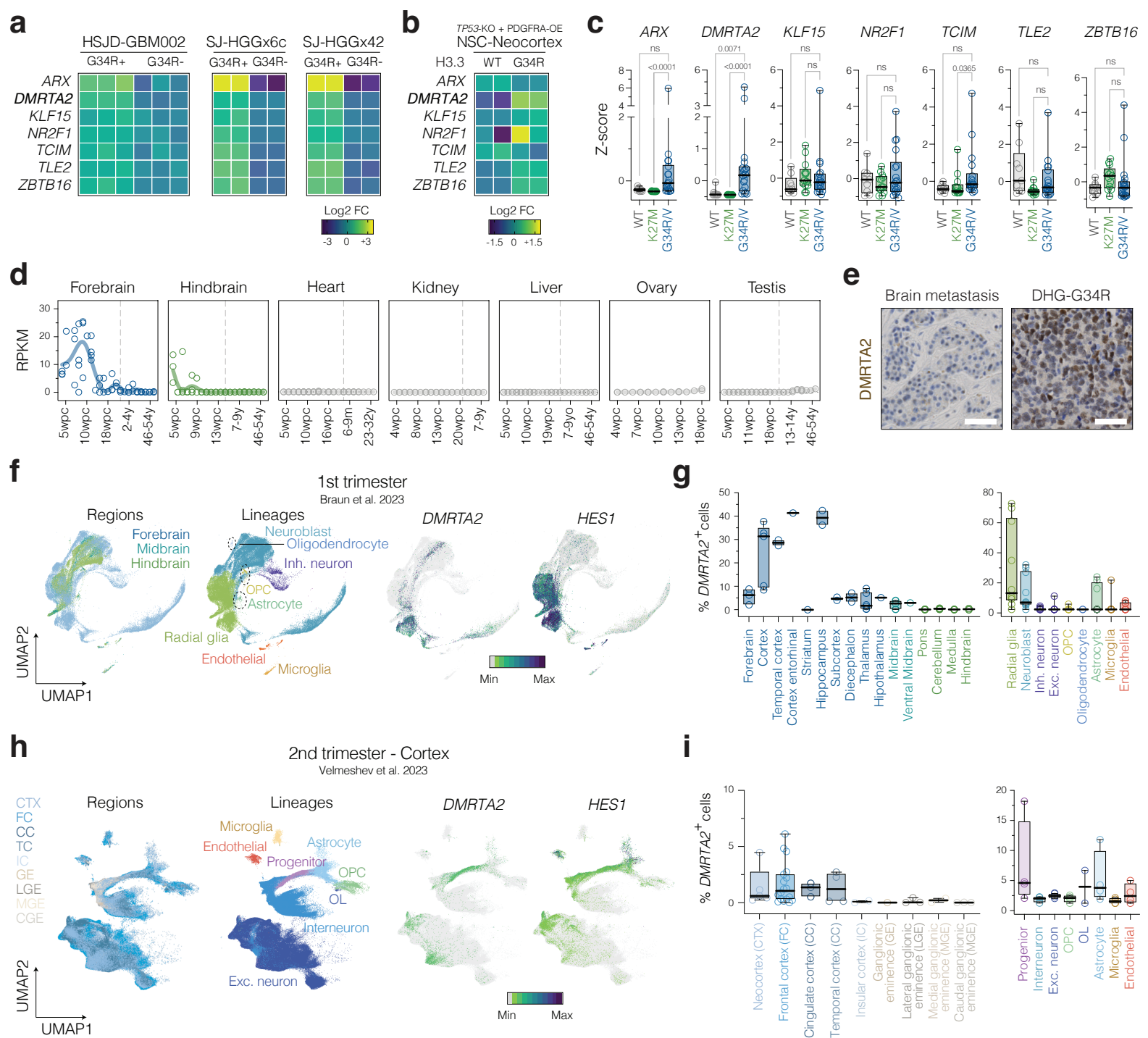

Supplement: Supplementary file 1 — Figure S1: Extended characterization of DMRTA2 expression across developmental and tumour contexts. (a) Heatmaps showing log2 fold‐change of candidate transcription factors following H3.3‐G34R correction in three independent patient‐derived isogenic H3G34‐mutant glioma models (HSJD‐GBM002, SJ‐HGGx6c and SJ‐HGGx42), highlighting DMRTA2 among the shared downregulated genes. (b) Heatmaps showing log2 fold‐change of the same candidate transcription factor genes in an engineered forebrain neural stem cell model (4) harbouring TP53 knockout (TP53‐KO), PDGFRA overexpression (PDGFRA‐OE) and either wild‐type or G34R‐mutant H3.3 constructs. (c) Z‐score expression of the seven candidate genes across WT, H3K27M and H3G34R/V paediatric high‐grade gliomas (2). Bars represent mean ± standard deviation. Statistical significance was assessed using one‐way ANOVA followed by Tukey's multiple comparisons test. Pairwise comparisons are indicated. n.s., not significant. (d) Scatter plots showing DMRTA2 expression across human tissues and developmental stages (12), demonstrating enrichment in the foetal forebrain relative to other organs. Horizontal dashed lines indicate birth. (e) Representative immunohistochemistry for DMRTA2 in a brain metastasis control and in a DHG‐G34R tumour biopsy. (f) 2D projection of first‐trimester human brain single‐cell RNA‐seq dataset (15), annotated by region and lineage, with DMRTA2 and HES1 expression overlaid. (g) Quantification of the percentage of DMRTA2‐expressing cells across first‐trimester brain regions and lineages as shown in panel f. (h) 2D projection of second‐trimester cortical single‐cell RNA‐seq dataset [14], annotated by region and lineage, with DMRTA2 and HES1 expression overlaid. (i) Quantification of the percentage of DMRTA2‐expressing cells across cortical regions and cellular lineages as shown in panel h. [file JCMM-30-e71302-s003.pdf]
