# Supplementary material for: Forebrain Lineage Factor DMRTA2 Is Not Required for Maintenance of H3G34‐Mutant Paediatric High‐Grade Gliomas
Source: J Cell Mol Med. 2026 Jul 25;30(14):e71302. doi: 10.1111/jcmm.71302 (PMC13401718; doi:10.1111/jcmm.71302)
Supplement: Supplementary file 2 — Figure S2: Extended characterision of DMRTA2 knockouts in patient derived DHG‐G34 cell lines. (a) Schematic of the DMRTA2 locus showing the position of the CRISPR/Cas9 sgRNA target sites, PCR amplification and Sanger sequencing primers. Blue arrowheads indicate the predicted Cas9 cleavage sites. Representative forward and reverse Sanger sequencing chromatograms from sgDMRTA2‐A‐ and sgDMRTA2‐B‐transduced cells demonstrate mixed nucleotide peaks arising immediately downstream of the predicted Cas9 cleavage sites, consistent with CRISPR‐mediated indel formation at the targeted locus. (b) IHC analysis confirming loss of DMRTA2 protein expression in SJ‐HGGx42 cells transduced with DMRTA2 shRNA‐B. Scale bar, 20 μm. (c) Representative bioluminescence imaging of orthotopic xenografts derived from SJ‐HGGx43 cells transduced with sgAAVS1 control and sgDMRTA2‐B. (d) Quantification of in vivo tumour burden by bioluminescence imaging in HSJD‐GBM002 orthotopic xenografts 1 month after intracranial transplantation. Data represent mean ± standard deviation. Statistical significance was assessed using an unpaired two‐tailed Student's t‐test with Welch's correction. n = 4 mice per group. (e) Right, Kaplan–Meier survival analysis of tumour‐bearing mice. μm. n = 4 animals per group. [file JCMM-30-e71302-s001.pdf]

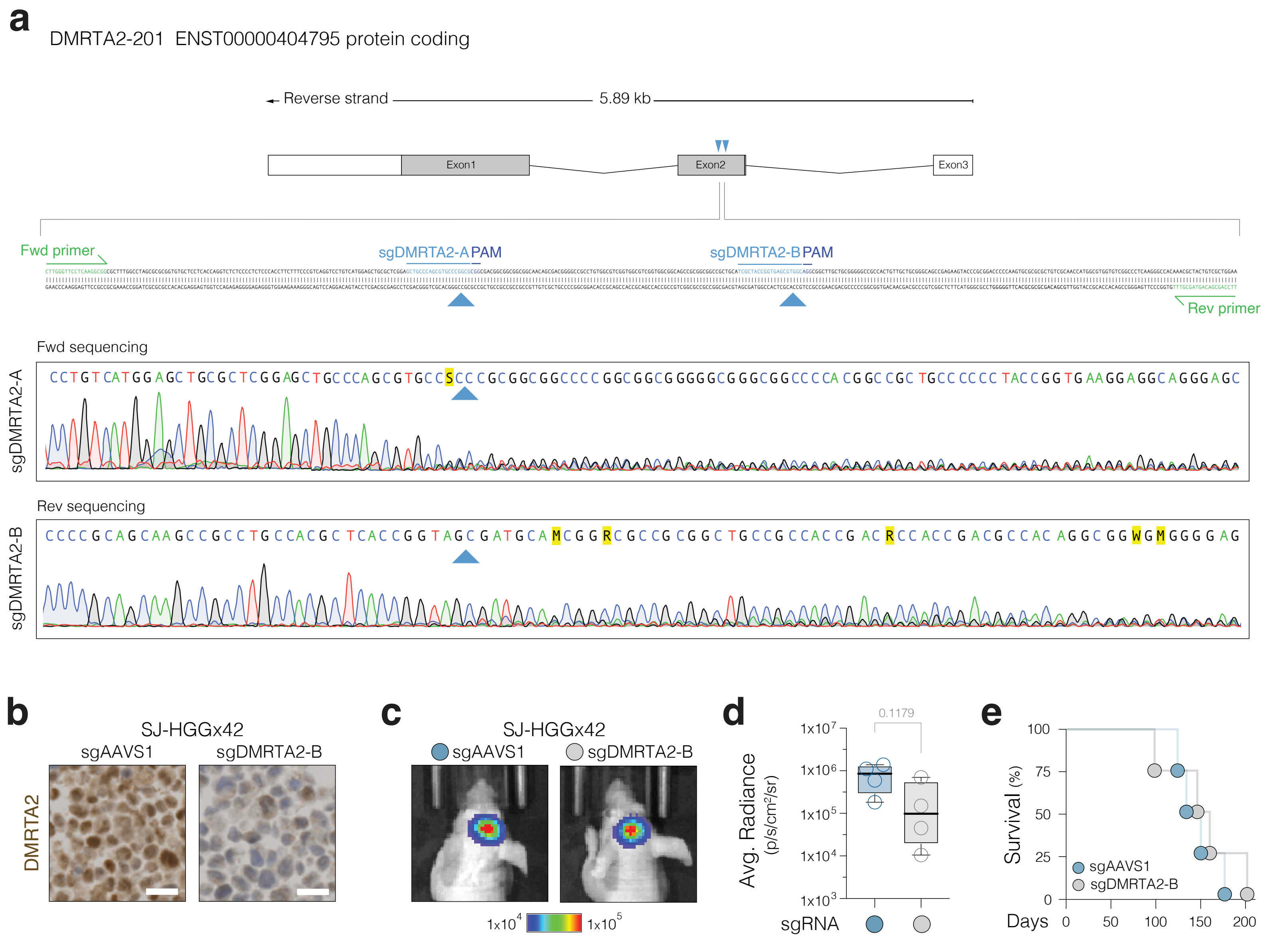
a
DMRTA2-201 ENST00000404795 protein coding
Reverse strand
5.89 kb
Exon1
Exon2
Exon3
Fwd primer
sgDMRTA2-A PAM
sgDMRTA2-B PAM
Rev primer
Fwd sequencing
sgDMRTA2-A
Rev sequencing
sgDMRTA2-B
b
SJ-HGGx42
sgAAVS1
sgDMRTA2-B
DMRTA2
c
SJ-HGGx42
sgAAVS1
sgDMRTA2-B
1x10^4
1x10^5
d
0.1179
Avg. Radiance (p/s/cm²/sr)
1x10^7
1x10^6
1x10^5
1x10^4
1x10^3
sgRNA
e
Survival (%)
100
75
50
25
0
sgAAVS1
sgDMRTA2-B
Days
0
50
100
150
200
